# Myoglobin regulates fatty acid trafficking and lipid metabolism in mammary epithelial cells

**DOI:** 10.1371/journal.pone.0275725

**Published:** 2022-10-12

**Authors:** Julia Armbruster, Mostafa A. Aboouf, Max Gassmann, Angela Egert, Hubert Schorle, Veit Hornung, Tobias Schmidt, Jonathan L. Schmid-Burgk, Glen Kristiansen, Anne Bicker, Thomas Hankeln, Hao Zhu, Thomas A. Gorr

**Affiliations:** 1 Institute of Veterinary Physiology, Vetsuisse Faculty, University of Zurich, Zurich, Switzerland; 2 Department of Biochemistry, Faculty of Pharmacy, Ain Shams University, Cairo, Egypt; 3 Zurich Center for Integrative Human Physiology, University of Zurich, Zurich, Switzerland; 4 Institute of Pathology, Department of Developmental Pathology, University Hospital Bonn, Bonn, Germany; 5 Gene Center and Department of Biochemistry, Ludwig-Maximilians-Universität München, Munich, Germany; 6 Institute of Clinical Chemistry and Clinical Pharmacology, University and University Hospital Bonn, Bonn, Germany; 7 Roche Pharmaceutical Research and Early Development, Roche Innovation Center Zurich, Schlieren, Switzerland; 8 Institute of Pathology, University Hospital Bonn, Bonn, Germany; 9 Institute of Organismic and Molecular Evolution, Molecular and Genome Analysis, Johannes Gutenberg University, Mainz, Germany; 10 Department of Clinical Laboratory Sciences, University of Kansas Medical Center, Kansas City, Kansas, United States of America; 11 Department of Biochemistry and Molecular Biology, University of Kansas Medical Center, Kansas City, Kansas, United States of America; Laurentian University, CANADA

## Abstract

Myoglobin (MB) is known to bind and deliver oxygen in striated muscles at high expression levels. MB is also expressed at much reduced levels in mammary epithelial cells, where the protein´s function is unclear. In this study, we aim to determine whether MB impacts fatty acid trafficking and facilitates aerobic fatty acid ß-oxidation in mammary epithelial cells. We utilized MB-wildtype versus MB-knockout mice and human breast cancer cells to examine the impact of MB and its oxygenation status on fatty acid metabolism in mouse milk and mammary epithelia. MB deficient cells were generated through CRISPR/Cas9 and TALEN approaches and exposed to various oxygen tensions. Fatty acid profiling of milk and cell extracts were performed along with cell labelling and immunocytochemistry. Our findings show that MB expression in mammary epithelial cells promoted fatty acid oxidation while reducing stearyl-CoA desaturase activity for lipogenesis. In cells and milk product, presence of oxygenated MB significantly elevated indices of limited fatty acid ß-oxidation, i.e., the organelle-bound removal of a C2 moiety from long-chain saturated or monounsaturated fatty acids, thus shifting the composition toward more saturated and shorter fatty acid species. Presence of the globin also increased cytoplasmic fatty acid solubility under normoxia and fatty acid deposition to lipid droplets under severe hypoxia. We conclude that MB can function in mammary epithelia as intracellular O_2_-dependent shuttle of oxidizable fatty acid substrates. MB’s impact on limited oxidation of fatty acids could generate inflammatory mediator lipokines, such as 7-hexadecenoate. Thus, the novel functions of MB in breast epithelia described herein range from controlling fatty acid turnover and homeostasis to influencing inflammatory signalling cascade. Future work is needed to analyse to what extent these novel roles of MB also apply to myocytic cell physiology and malignant cell behaviour, respectively.

## Introduction

Globins are hemoproteins capable of binding various gas molecules, such as O_2_, CO, NO^.^ and H_2_S [1]. The globin family in animals includes phylogenetically conserved members, namely, hemoglobin, myoglobin (MB), cytoglobin (CYGB), neuroglobin (NGB) and androglobin (ADGB) [2]. Recently, emerging evidence suggested that MB and CYGB also bind fatty acids (FAs) *in vitro*. In CYGB, FA binding stabilizes the penta-coordination and promotes its peroxidase activity [3], which can also be induced by various anionic lipids [4].

MB is known to deliver and store oxygen in striated muscles, where MB concentration ranges from high micromolar in terrestrial mammals to millimolar in deep-diving mammals [5].

The ectopic occurrence of MB in tissue beyond skeletal/myocardial muscle was first reported in the liver and other organs of hypoxia-tolerant fish models [6–8]. More recently, we and others found that MB is expressed in several cancer cell lines and epithelial tumors [9–11] and in mouse brown adipose tissue [12, 13]. Muscles and breast epithelia express MB of identical amino acid sequences but at different levels. Although the level of MB protein expression is 350 times higher in human breast cancer samples than in matching normal breast tissues [10], MB expression level is far lower in breast epithelia than muscle cells, suggesting that MB fulfils functions other than the classic O_2_ binding and transportation.

*In vitro*, oxygenated MB (MBO_2_) binds long-chain FAs, palmitate (C16:0, saturated) and oleate (C18:1n9, monounsaturated), with physiological constants [14, 15]. MBO_2_ also binds medium- to long-chain FAs (C12:0—C18:1) and acylcarnitines in a 1:1 stoichiometry, potentially regulating acylcarnitine and FA pools in MB-rich tissues [16]. Studies in mice with targeted MB gene knockout (MBko) suggest that MB is involved in the control of FA turnover and homeostasis in myocytes and non-muscle cells. When compared to wildtype (MBwt) counterpart, the heart of MBko mice switched substrate utilization in energy production from highly aerobic FA ß-oxidation to relatively O_2_-sparing glucose oxidation [17]. Cardiomyocytes in older MBko mice develop a lipid overload pathology that impairs heart function [18], suggesting that MB in cardiomyocytes mediates not only O_2_ supply but also FA turnover. This conclusion is supported by our recent study of mouse brown adipose tissue (BAT), where MB expression stimulates oxidative metabolism along with uncoupled respiration. In addition, presence of MB in BAT also influences the free fatty acids pool towards a palmitate-enriched composition and shifts the lipid droplet (LD) equilibrium towards higher counts of smaller droplets, thereby supporting the browning phenotype [12].

MB exists at low levels (nanomolar to micromolar) in healthy breast parenchyma, breast cancer cell lines, 40% of invasive ductal mammary carcinomas, and 70% of invasive breast cancers [19]. In the mammary glands of healthy women and female mice, MB is expressed exclusively in lipid-secreting inner luminal cells of the milk duct epithelium [10, 19], suggesting a potential role in FA trafficking and LD homeostasis *in vivo* [15, 20]. We hypothesize that MB impacts FA metabolism in mammary epithelial cells in a manner similar to that in cardiomyocytes and BAT.

To test this hypothesis, we analysed FA composition of the lipids from MBwt and MBko mouse milk and the lipids from human breast cancer cells with or without MB expression. We also examined distribution of FA and LDs among cellular compartments under various oxygen tensions. We show here that (1) oxygenated MB (MBO_2_) supports FA oxidation, not desaturation, (2) MB expression under normoxia (MBO_2_) promotes cytoplasmic solubility of FAs, (3) MB expression under severe hypoxia (deoxy-MB) deposits FAs to LDs in conjunction with oxygen unloading, and (4) lysosomal association of LDs appears to be MB-dependent and stimulated by severe hypoxia. Our findings shed novel insights to the functional involvement of MB in regulating FA metabolism in mammary epithelial cells during health and disease.

## Materials and methods

### MB knockout mice and milk extraction

All animal procedures were approved by the LANUV-NRW in Recklinghausen, Germany (84–02.04.2014.A453) and were performed in accordance with the German animal protection laws (in accordance with the NIH Publications No. 8023, revised 1987) and complies with the ARRIVE guidelines. Systemic MB knockout mice (NMRI strain background) were obtained via embryo transfer [21]. Gene ablation was achieved by deleting exon 2 that encodes amino acid residues for heme binding via homologous recombination. Mice were bred at the Laboratory Animal Service Centre, University of Zurich, Zurich, Switzerland, and only female adults were used for our experiments. They were housed at 22±5°C in a 12 h light/dark cycle and fed with a standard rodent chow diet as previously described [12].

MBwt and MBko female mice were milked 3–5 days after labour. For milk extraction, mice were anesthetized using Ketanest/Rompun. To stimulate lactation, mice were injected i.m. with oxytocin and the breast tissue was massaged, and 300–500 μl milk was extracted per mouse with the use of a vacuum pump for FA profiling.

### Fatty acid profiling

Mouse milk and MDA-MB468 cell pellets (collected after a 72-hour incubation under various oxygen tensions) were frozen and shipped on dry ice to the University of Kansas Medical Center (Kansas City, USA) for FA profiling with an established protocol [22]. Prior to lipid extraction, a fixed amount of authentic unnatural heptadecanoic acid (C17:0) corresponding to each lipid class of interest was added as internal reference for quantification. Total lipids were extracted using the Folch protocol, i. e. chloroform/methanol of 2:1 supplemented with 0.1% butylhydroxytoluene to prevent lipid oxidation. Major subgroups, including triglycerides (TGs), diglycerides (DGs), free fatty acids (FFAs), and total phospholipids (PLs), were separated via thin layer chromatography on silica gel plates using a solvent system of hexane: diethyl ether: acetic acid (80:20:1). Upon visualization under UV with 2,5-Bis(5-tert-butyl-benzoxazol-2-yl)thiophene staining, each fraction was recovered and subjected to transmethylation with boron trifluoride in methanol (Benzene also added for TG and total lipids). The resulting fatty acid methyl esters (FAMEs) were analysed on a Varian GC450 system (Palo Alto, CA), with a Varian FAME Select CP 7420 100m 0.25-mm column. FAME mix standards (Supelco 37 component from Sigma, 47885U) were used to confirm the identity of FAMEs. Flame ionization detection was used to quantify each FAME, and the absolute amount was calculated against that of C17:0.

### Generation of MBko cells

MBko MCF7 single-cell colonies were generated using the CRISPR/Cas9 method to disrupt exon 2 of the MB gene that encodes the heme-binding pocket. The following sgRNA sequences with the lowest rate of off-target effect score were designed and combined with 4-base overhangs (in bold) to facilitate ligation to CRISPR/Cas9 plasmids: F: 5’-**CACCg**GGACGAGATGAAGGCGTCTG-3’ and R: 5’-**AAAC**CAGACGCCTTCATCTCGTCCc-3’. Successful targeting of the two MB alleles in each MCF7 clone was confirmed by polymerase chain reaction (PCR)-based genotyping with 5’-TGGGAAGACAGGGAGCTAAA-3’ and 5’-GCTCTGCCATTATCCACCTC-3’ as primers and by subsequent Sanger sequencing. Bioinformatic analysis of the sequence chromatograms using the TIDE tool (https://tide.nki.nl) detected a 5 bp deletion on one allele and a 2 bp insertion on the other, with both modifications leading to a premature stop codon (S1A Fig). The absence of MB protein was confirmed by Western Blotting (S1B Fig) using specific anti MB antibody (FL-154, cs-25607, Santa Cruz Biotechnology, 1:200).

In addition to MCF7, MDA-MB468 cells were chosen due to their pronounced basal and hypoxia-inducible expression of MB [10, 19] and a different p53 background, i.e., the R273H hemizygous transcriptionally active mutation with strong gain-of-function effects on breast cancer survival in contrast to the wildtype p53 status in MCF7 cells [23, 24]. MBko MDA-MB468 cells were generated using the TALEN (transcription activator-like effector nuclease) approach to introduce frame-shift mutations to exon 2 of the MB gene. A pair of TALEN expression plasmids was assembled using ligation-independent cloning [25], targeting the 5’-TGCTCACCGCCCTGGGTGGCATCCTTAAGAAGAAGGGGCATCATG-AGGCA-3’ sequences within the human MB gene (codons 70–85, encoding for LTALGGILKKKGHHEA). Genome-editing activity of the TALEN pair was confirmed by transient plasmid transfection into HEK 293T cells and subsequent targeted deep sequencing [25]. MDA-MB468 cells were transfected with TALEN expression plasmids. Mutations within individual MB alleles in single-cell clones were determined by Sanger sequencing. The absence of MB protein expression in MBko cells was further confirmed by immunoblot analysis (S1C Fig).

### Cell culture and hypoxia exposure

Human breast cancer cell lines MCF7 (HTB-22) and MDA-MB468 (HTB-132) were obtained from ATCC (Manassas, VA, USA) and cultured at 37°C in EMEM (ATCC 30–2003) and DMEM (Gibco 119600430–2008, supplemented with 5 mM GlutaMax, Gibco 35050061), respectively. Normoxia was reached with room air in a Nuaire incubator (NU-5810E; 60% humidity, 5% CO_2_), whereas various hypoxic conditions (5%, 1% or 0.2% O_2_) were applied in a hypoxic glove box (Coy Laboratory Ltd., Leeds, UK; 60% humidity, 5% CO_2_).

### Cell labelling and immunocytochemistry

Cells were seeded onto round cover slips and incubated under normoxia or severe hypoxia (0.2% O_2_) prior to further processing as described below. After the corresponding staining of the cells, DAPI was added in a 1:1000 dilution for 5 min to stain the nucleus of cells. The cover slips were then mounted on ProLong Gold Antifade Mountant (Thermo Fisher Scientific, P36930) prior to imaging.

### BODIPY staining

BODIPY FL C16, a fluorescent palmitate derivative (Thermo Fisher Scientific, D3821), was used to treat cells at a working concentration of 5 μM for 30 min prior to normoxia (room air) or severe hypoxia (0.2% O_2_). After 72 hrs, cells were either fixed in 4% paraformaldehyde (PFA, freshly prepared in PBS) prior to mounting or used for further co-staining (see below).

### Antibody staining

After PFA fixation, cells were permeabilized with 0.1% Triton-X-100 in PBS during 5 min. For blocking, cells were incubated with 10% goat serum in PBS for 1h at room temperature. Primary antibodies, diluted in 3% goat serum in PBS, against MB (FL-154, cs-25607, Santa Cruz Biotechnology, 1:500), CD36 (ab133625, Abcam, 1:50), or PMP70 (ab3421, Abcam, 1:500), were incubated with cells at 4°C overnight. Upon PBS washing (3 x 10 min), dye-coupled secondary antibodies, diluted 1:1000 in 3% goat serum in PBS, were incubated with the cells for 45 min at room temperature.

### Lysosome staining

After 72 hrs incubation under room air (Nx) or 0.2% O_2_ (Hx), cells were stained with Cytopainter red (Lysosomal Staining Kit- Red Fluorescence, ab112137) for additional 1 hr under Nx or Hx. Cells were then fixed in 4% PFA and mounted as described above. For fluorescence-activated cell sorting (FACS), cells were collected and resuspended in PBS to measure the intensity of lysosomal staining (Gallios, Beckman Coulter, AU29614).

### Mitochondrial labelling

Cells were treated with MitoTracker Deep red FM (M22426, ThermoFisher Scientific) according to the manufacturers protocol. A working concentration of 300 nM MitoTracker diluted in cell culture medium was used to incubate cells for 30 min.

### Lipid droplet staining

Cells were incubated for 10 min with a lipid droplet marker, Nile red (N1142, ThermoFisher Scientific), 0.5 μg/ml in PBS as described in [26]. The slides were analysed using a fluorescent microscope (Zeiss Imager Z2) coupled to an 8-bit CCD camera.

### Statistical analysis

Results are expressed as mean ± SD of at least 3 independent experiments (n indicated in figure legend). Statistical analysis was performed using GraphPad Prism 6 (GraphPad Software, USA). Normal distribution of data population was tested using Shapiro-Wilk test. Significance was determined by Students t-test (parametric data). P value ≤ 0.05 was considered significant.

## Results

### MB expression impacts FA profiles of mouse milk and human breast cancer cells

**Fig 1:**
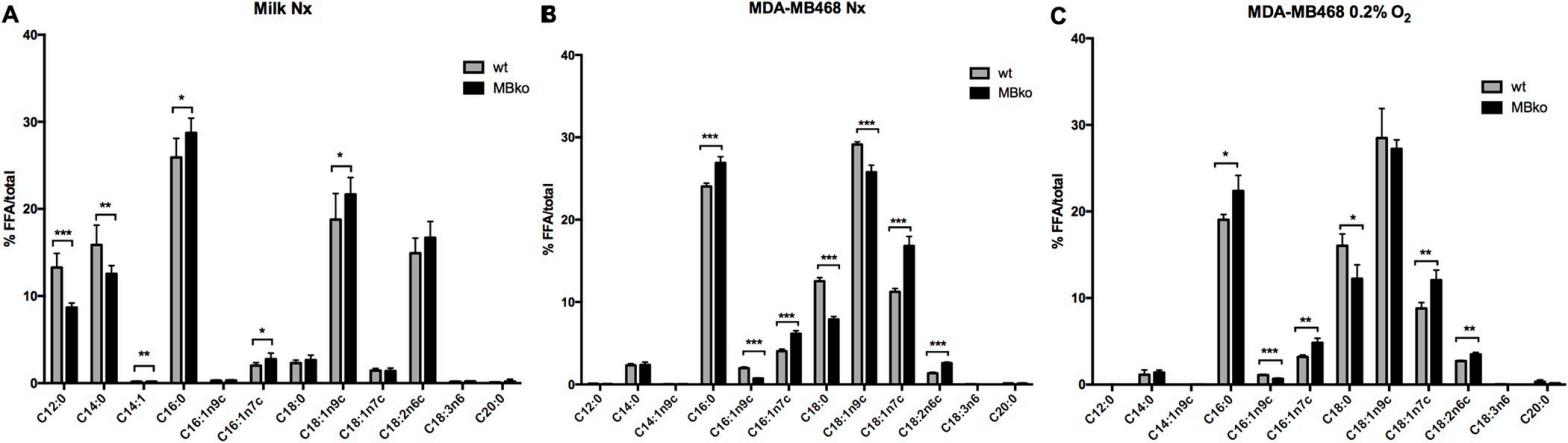
MB-dependent fatty acid profiles. Fatty acid profiles (C12-C20) of total lipids of the mouse milk (**A**), MDA-MB468 breast cancer cells cultured under normoxia (Nx, **B**) and severe hypoxia (0.2% O_2_, **C**). In all graphs, MBwt as grey bars vs MBko black. FA levels are depicted as “% of FA / total FA pool”. Students t-test was used for statistics (n = 4–8); mean ± SD. *p≤0.05, **p≤0.01, ***p≤0.001. The axis is labelled FFA.

We first examined the effect of MB on FA metabolism in mammary epithelial cells by performing FA profiling from breast milk of MBwt and MBko mice. We observed that triglycerides (TG) make up approximately 90% of the total lipids (S2A Fig) and that the major FAs contained in milk are laureate (C12:0), myristate (C14:0), palmitate (C16:0), oleate (C18:1n9c), and linoleate (C18:2n6c) (Fig 1A), all of which confirming previous findings by others [27]. As shown in Fig 1A, MB expression in mammary epithelial cells resulted in a higher percentage of medium-chain FAs (C12:0, C14:0, and C14:1) and a lower percentage of long-chain FAs (C16:0, C16:1n7c, and C18:1n9c) in the milk of MBwt mice when compared to MBko counterparts.

We next performed FA profiling for human mammary epithelial adenocarcinoma MDA-MB468 cells that express MB at high levels [10]. Applying the Hill equation (saturation = pO_2_ / (pO_2_ + P_50_)) with its oxygen loading/unloading hyperbola, MB exists mostly in its oxygenated state (MBO_2_) under normoxia (98%) and moderate hypoxia (i.e. 5% O_2_ (93%) and 1% O_2_ (71%); % O_2_ saturation given in parentheses, based on the MB P_50_ of 3mmHg) but is predominantly deoxygenated under severe hypoxia (0.2% O_2_ (33%)) [28]. We therefore cultured MBko and MBwt cells under normoxia and severe hypoxia to examine the impact of MB and its oxygenation status on cellular FA profiles. Phospholipids (PL) make up most of the total lipids (S2B Fig). FA profiles mostly consisted of oleate (C18:1n9c), palmitate (C16:0), stearate (C18:0) and palmitoleate (C16:1n7c) (Fig 1B), with far less medium-chain FAs (12–14 carbons) than in mouse milk. Under normoxia, MB expression in MDA-MB468 cells resulted in a lower percentage of C16:0, C16:1n7c and C18:1n7c, as well as a higher percentage of C18:0, C18:1n9c and C16:1n9c (Fig 1B). This alteration is different from the one observed in milk in which the latter species showed either the opposite trend (C18:1n9c) or no difference between MBko and MBwt cells (C18:0 and C16:1n9c) (Fig 1A).

Under severe hypoxia, MBwt and MBko cells (Fig 1C) exhibited a similar difference in FA profiles as seen under normoxia (Fig 1B). In both MBwt and MBko cells, the transition from normoxia to severe hypoxia reduced palmitate content while elevating stearate and oleate levels.

### MB expression antagonizes fatty acid desaturation

We examined the impact of MB expression on biochemical pathways that are essential to FA turnover, namely, fatty acid synthesis, elongation, oxidation, and desaturation [29]. We focused on the latter two processes because these reactions require molecular oxygen that is not involved in either fatty acid synthase or elongase catalyses.

**Fig 2:**
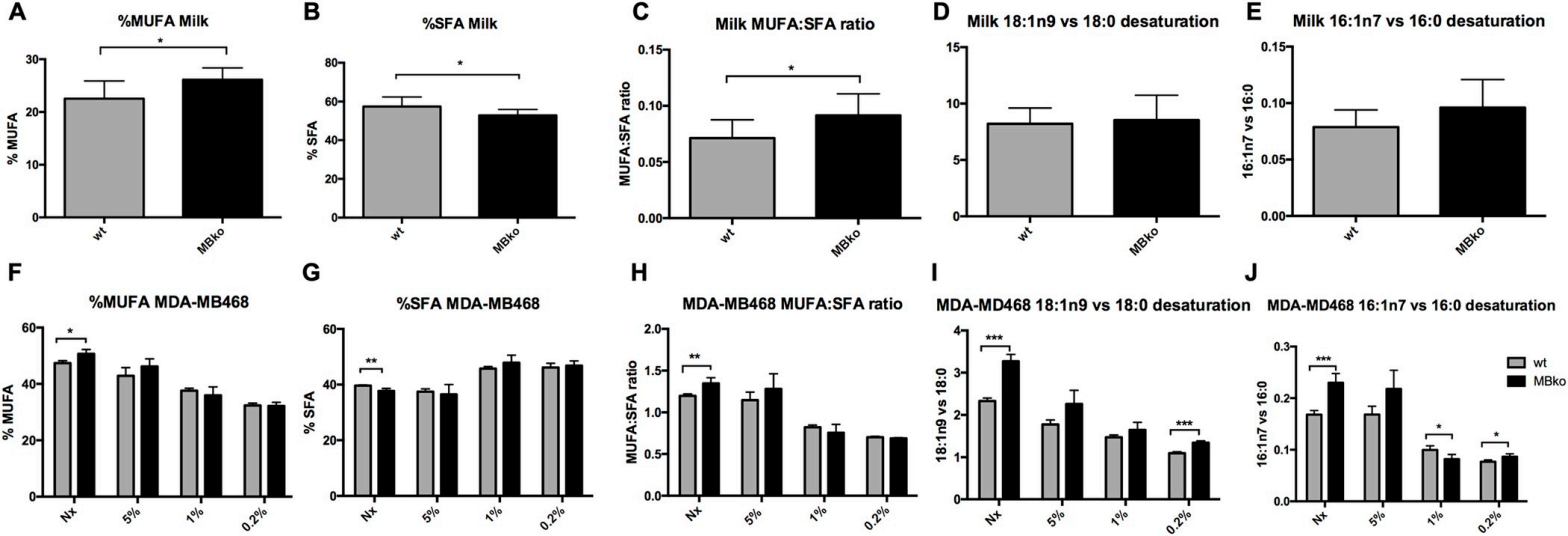
Impact of MB on indices of saturated and monounsaturated fatty acids. FA compositions of the mouse milk and MDA-MB468 cells were presented as the percent of major MUFAs (C16 and C18, n9 and n7 species; **A** and **F**, respectively) or SFAs (C12, C14, C16, C18, C20; **B** and **G**, respectively) among total FAs amount, and as the MUFA vs. SFA ratio (**C** and **H**, respectively). The delta-9 desaturation index was measured as oleate (C18:1n9) vs. stearate (C18:0) ratio (**D** and **I**, respectively) or palmitoleate (C16:1n7) vs. palmitate (C16:0) ratio (**E** and **J** respectively). MDA-MB468 cells were exposed to four O_2_ tensions for 72 hrs: Nx, 5% and 1% O_2_ (oxygenated MB), 0.2% O_2_ (deoxygenated MB). In all graphs, MBwt as grey bars versus MBko black. Students t-test was used for statistics (n = 4–8); mean ± SD. *p≤0.05, **p≤0.01, ***p≤0.001.

In mouse milk, MB expression resulted in a lower percentage of total MUFAs (monounsaturated fatty acids) and a higher percentage of total SFAs (saturated fatty acids) when compared to MBko counterparts (Fig 2A and 2B). The same was observed for MBwt MDA-MB468 cells under normoxia (Fig 2F and 2G). These changes, when combined, resulted in a reduced MUFA vs. SFA ratio in the MBwt mouse milk (Fig 2C) and the cells under normoxia (Fig 2H).

To examine the impact of MB to oxygen-dependent delta-9 desaturation, we further analysed FA profiles using C18:1n9 vs. C18:0 ratio, and C16:1n7 vs. C16:0 ratio, as indices of desaturation. No difference in these indices were observed in mouse milk (Fig 2D and 2E). However, the presence of MB in MDA-MB468 cells reduced desaturation indices under normoxia and severe hypoxia (Fig 2I and 2J). Decreasing desaturation indices in hypoxic cells probably reflect, regardless of the cells`MB status, the O_2_-dependence of these reactions (Fig 2I and 2J).

### MB expression promotes limited FA oxidation under normoxia and moderate hypoxia

To examine the impact of MB on oxygen-dependent long-chain FA oxidation, we further analysed indices of limited ß-oxidation: C14:0 vs. C16:0, and C16:1n9 vs. C18:1n9, i.e., the organelle-bound removal of a C2 moiety from either long-chain SFAs or MUFAs. These FA species were chosen due to a nearly exclusive conversion by oxidation.

**Fig 3:**
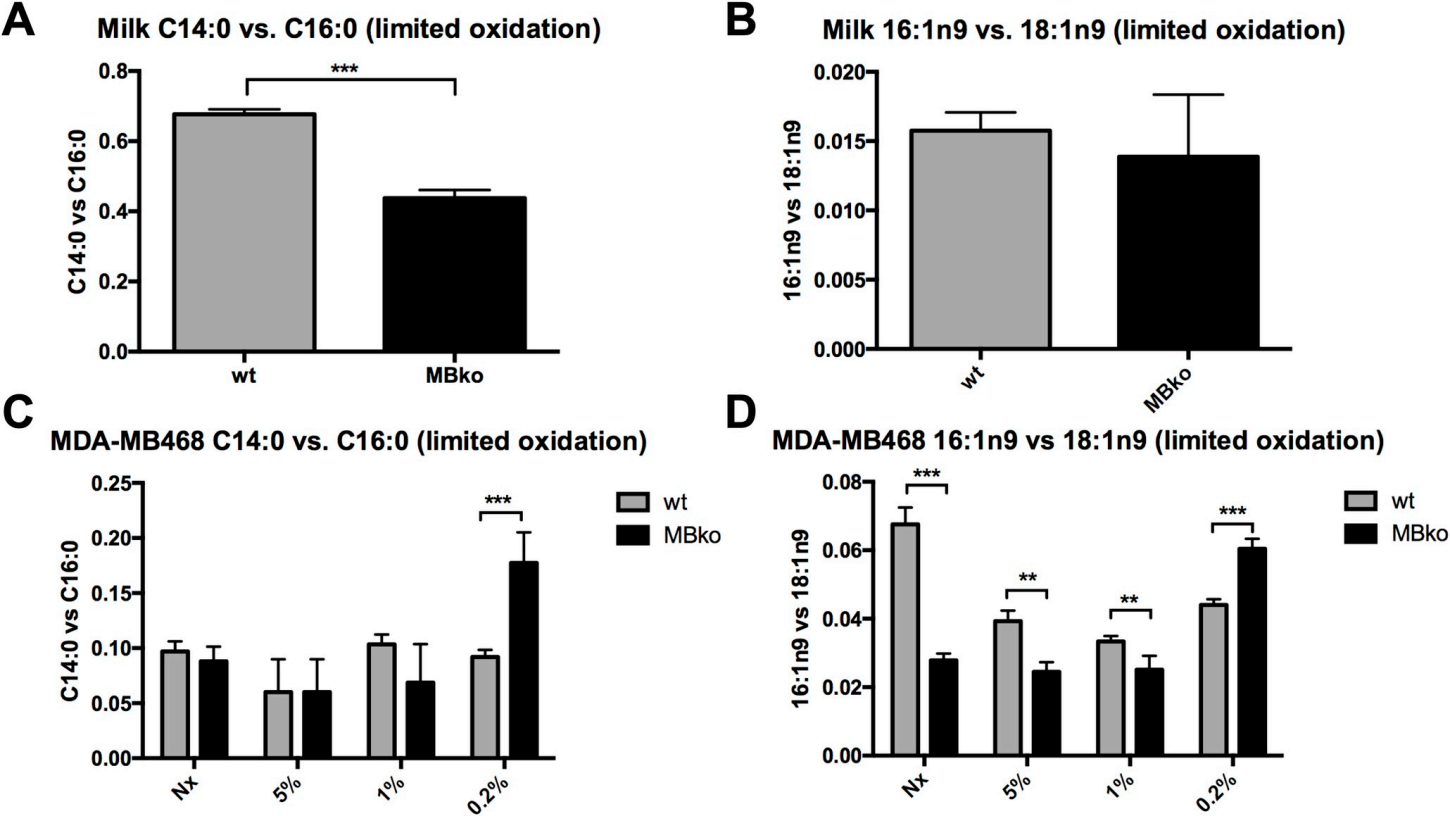
MB’s control of the limited oxidation of fatty acids. Limited oxidation was measured as C14:0 vs. C16:0 ratio or C16:1n9 vs. C18:1n9 ratio in mouse milk (**A, B**) and MDA-MD468 cells (**C, D**). In all graphs, MBwt as grey bars versus MBko black. MDA-MB468 cells were exposed to Nx, 5% O_2_, 1% O_2_ (oxygenated MB) or 0.2% O_2_ (deoxygenated MB). Students t-test was used for statistics (n = 4–8); mean ± SD. **p≤0.005, ***p≤0.001.

In the milk, MB expression significantly increased the C14:0 vs. C16 0 ratio by 1.6-fold (Fig 3A), whereas the C16:1n9c vs. C18:1n9c ratio was unaltered by the MB genotype (Fig 3B). In MDA-MB468 cells under normoxia, MB expression significantly increased C16:1n9c vs. C18:1n9c ratio by 2-fold (Fig 3D), but not the C14:0 vs. C16 0 ratio (Fig 3C). In other words, MB-proficient MDA-MB468 cells are more than twice as effective in conducting the limited oleate (C18:1n9c) → hexadecenoate (C16:1n9c) oxidation than MB-deficient counterparts. Moreover, these oxidation indices progressively declined under reducing oxygen tensions in both MBwt and MBko cells. Eventually, the index of limited oxidation flipped to a MBko > MBwt readout in severely hypoxic cells (0.2% O_2_), suggesting that accumulating deoxygenated MB might potentially act as an inhibitor of this reaction (Fig 3D). The fact that the C14:0 vs. C16:0 ratio was more pronounced in the milk, while the C16:1n9c vs. C18:1n9c ratio was higher in MDA-MB468 cells, might point to a different physiological importance of these FA´s for cellular lipids vs. secreted lipids in milk.

### MB expression impacts on intracellular fatty acid distribution

To further examine the impact of MB on FA metabolism in mammary epithelial cells, we determined intracellular localization of FAs in MBwt and MBko breast cancer cells by using BODIPY-FL-C16, a fluorogenic derivative of palmitate. In addition to MDA-MB468 cells, we also used human mammary epithelia carcinoma MCF7 cells that express MB at a lower level than MDA-MB468 [10]. MBwt and MBko cells of both lines were incubated with BODIPY and then subjected to either normoxia (Nx, air) or severe hypoxia (Hx, 0.2% O_2_), thereby allowing to discriminate the impact of oxygenated or deoxygenated MB, respectively.

**Fig 4:**
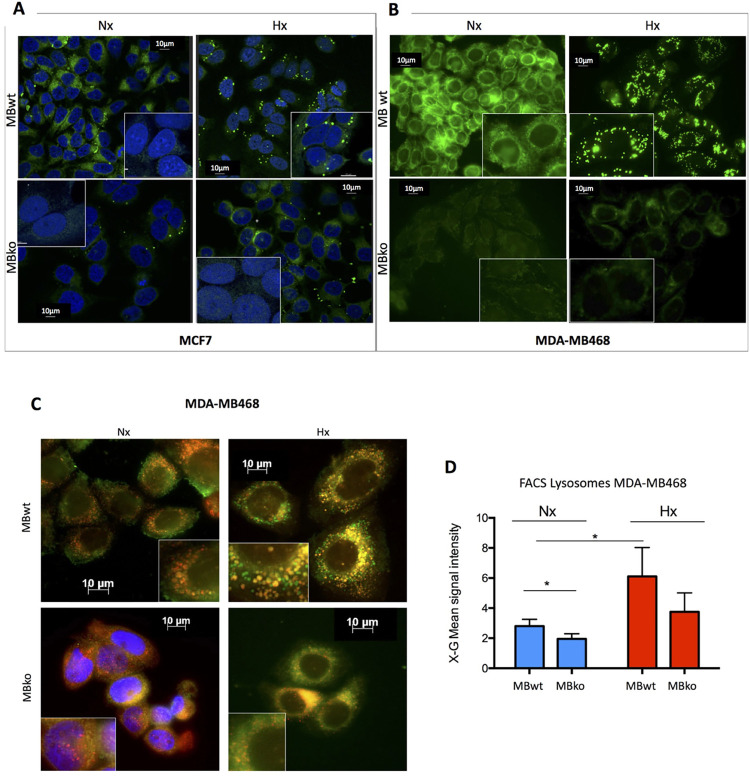
MB-based trafficking of fatty acids. MB in MCF7 (**A**) and MDA-MB468 (**B**) cells controls the transport of a fluorogenic palmitate derivative (BODIPY-FL C16) between cytoplasm and vesicular depots in an O_2_-dependent fashion. Cells were incubated with BODIPY (green) for 30 min before exposure for 72 hrs to normoxia (Nx, air, oxygenated MB) or severe hypoxia (Hx, 0.2% O_2_, deoxygenated MB). The nuclei of MCF7 cells were stained with DAPI (blue). The figures shown are the representative of 6 independent experiments. Scale bar: 10 μm. Magnification: 63x. (**C)** MBwt and MBko MDA-MB468 cells incubated under normoxia (Nx) or severe hypoxia (Hx) were co-stained with BODIPY and a lysosome marker (Cytopainter red). The figures shown are representative of 3 independent experiments. Scale bar: 10 μm. Magnification: 63x. MBko (Nx) cells were stained by DAPI for nuclei. (**D**) Lysosomal (Cytopainter red) staining in MDA-MB468 cells was analysed by FACS measurement with the signal intensity expressed as geometric mean (X-GMean). Students t-test was used for statistics (n = 5); mean ± SD. *p≤0.05.

In normoxic MCF7 and MDA-MB468 cells, MB expression improved the solubility of the palmitate derivative primarily in the cytoplasm near cell periphery (Fig 4A and 4B, respectively, top-left panels). In severely hypoxic MBwt cells, BODIPY-FL-C16 deposition coincided with vesicles (puncta) (Fig 4A and 4B, top-right panels), and the intensity of FA signals was weaker in MCF7 than MDA-MB468 cells. These signals became even weaker in MBko cells regardless of their oxygenation (Fig 4A and 4B, bottom panels).

### MB promotes fatty acid deposition to organelles upon unloading of bound oxygen

We further characterized MB-dependent vesicular FA deposition under severe hypoxia through co-staining with markers of subcellular compartments in MDA-MB468 cells. The BODIPY-labelled vesicles revealed an average size of 1.36 ± 0.46 μm (mean ± SD, 20 independent measurements). Therefore, we focused on organelles and vesicles of similar sizes, including mitochondria (0.5–1 μm), peroxisomes (0.1–1 μm), lysosomes (0.5–1.5 μm) and LDs (20 nm—100 μm) [30, 31]. In MBwt cells, we observed partial overlay between BODIPY and the lysosome marker Cytopainter red (Fig 4C), but not peroxisomes marker PMP70, mitochondrial MitoTracker Red, or FA importer CD36, which is a facilitator for lipid droplet formation (S3A–S3C Fig, respectively).

To further investigate the association between MB status and lysosomal FA deposition, we used fluorescence-activated cell sorting (FACS) to quantify the intensity of lysosome staining in MDA-MB468 cells. MBwt cells exhibited a small but significant increase in the signals relative to MBko cells under normoxia, while severe hypoxia induced a ~ 2-fold increase in the lysosomal signals in both MBwt and MBko cells (Fig 4D). In comparison, MCF7 cells exhibited no detectable signal overlay between BODIPY and lysosomes under severe hypoxia, nor genotypic difference in the lysosomal signals under normoxia and severe hypoxia (S3D and S3E Fig).

### MB expression promotes LD formation and FA storage in severely hypoxic cells

**Fig 5:**
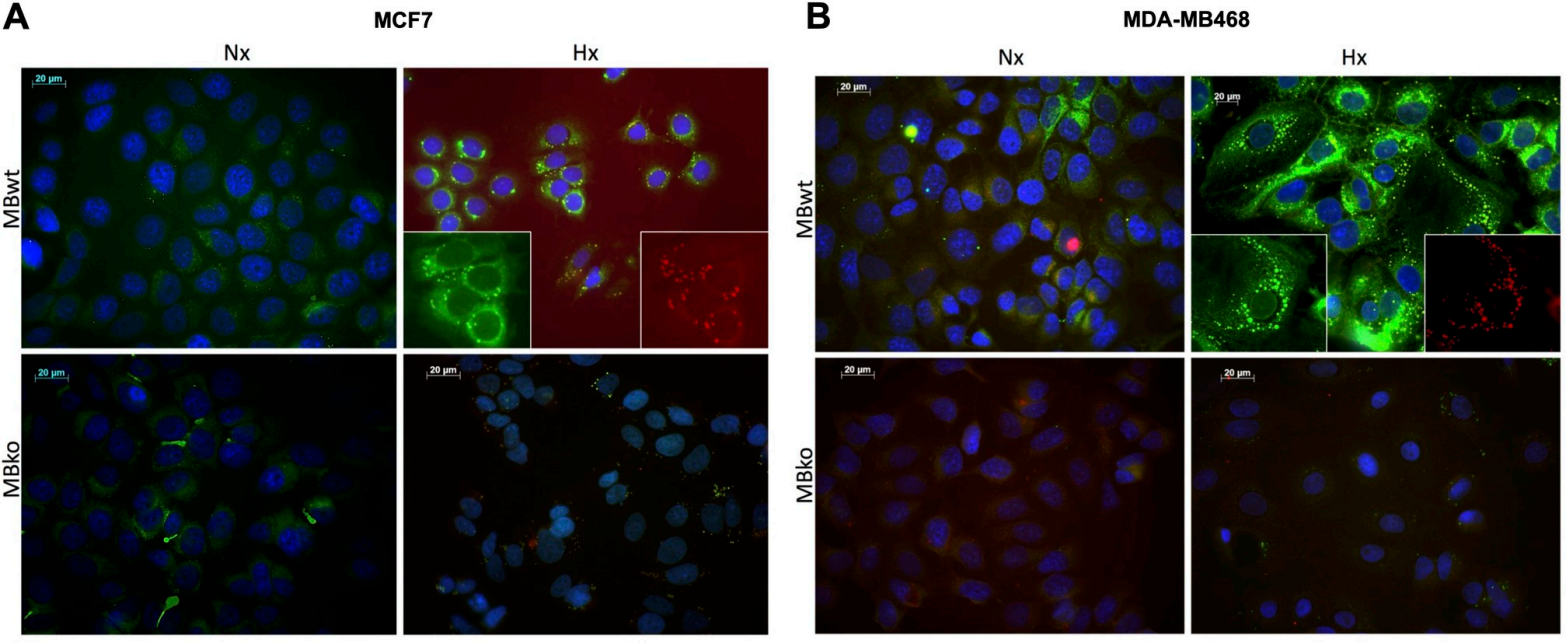
Lipid droplet homeostasis and MB. MB regulates lipid droplet homeostasis in an O_2_-dependent fashion. MCF7 (**A**) and MDA-MB468 (**B**) cells were incubated with BODIPY-FL C16 (green) for 30 min before exposure for 72 hrs to normoxia (Nx, oxygenated MB) or severe hypoxia (Hx, 0.2% O_2_, deoxygenated MB). Cells were then fixed and co-stained with Nile red for lipid droplets and DAPI (blue) for nuclei. The figures shown are representative of 3 independent incubations. Scale bar: 20 μm. Magnification: 63x. Small inserts represent selected areas in higher magnification.

We examined the potential FA deposition to LDs in MCF7 and MDA-MB468 cells by co-staining with BODIPY (FA) and Nile red (LDs). Shown in Fig 5A and 5B, the intensity of LD signals (red) markedly increased in severely hypoxic MBwt cells, possibly due to the enlargement of these vesicles. MB deficiency in both MCF7 and MDA-MB468 cells diminished the appearance of green (FA) and red (LD) signals under normoxia and severely hypoxia. The FA-LD colocalization was more clearly detectable in separately captured images (green vs red) than superimposed ones in which FA signal was much stronger than LDs (small inserts of Fig 5A and 5B). These data suggest that MB expression in mammary epithelial cells may regulate LD formation in response to varying oxygenation conditions, thus shifting the cellular phenotype from a “plurilocular”, highly oxidative (browning) state under normoxia and moderate hypoxia to an “oligolocular”, FA-storing (whitish) state during periods of oxygen starvation. MB appears to deposit oxygen and FA substrates in parallel to vesicular bodies once oxygen levels have dropped sufficiently low to trigger the O_2_-unloading of this globin.

## Discussion

**Fig 6:**
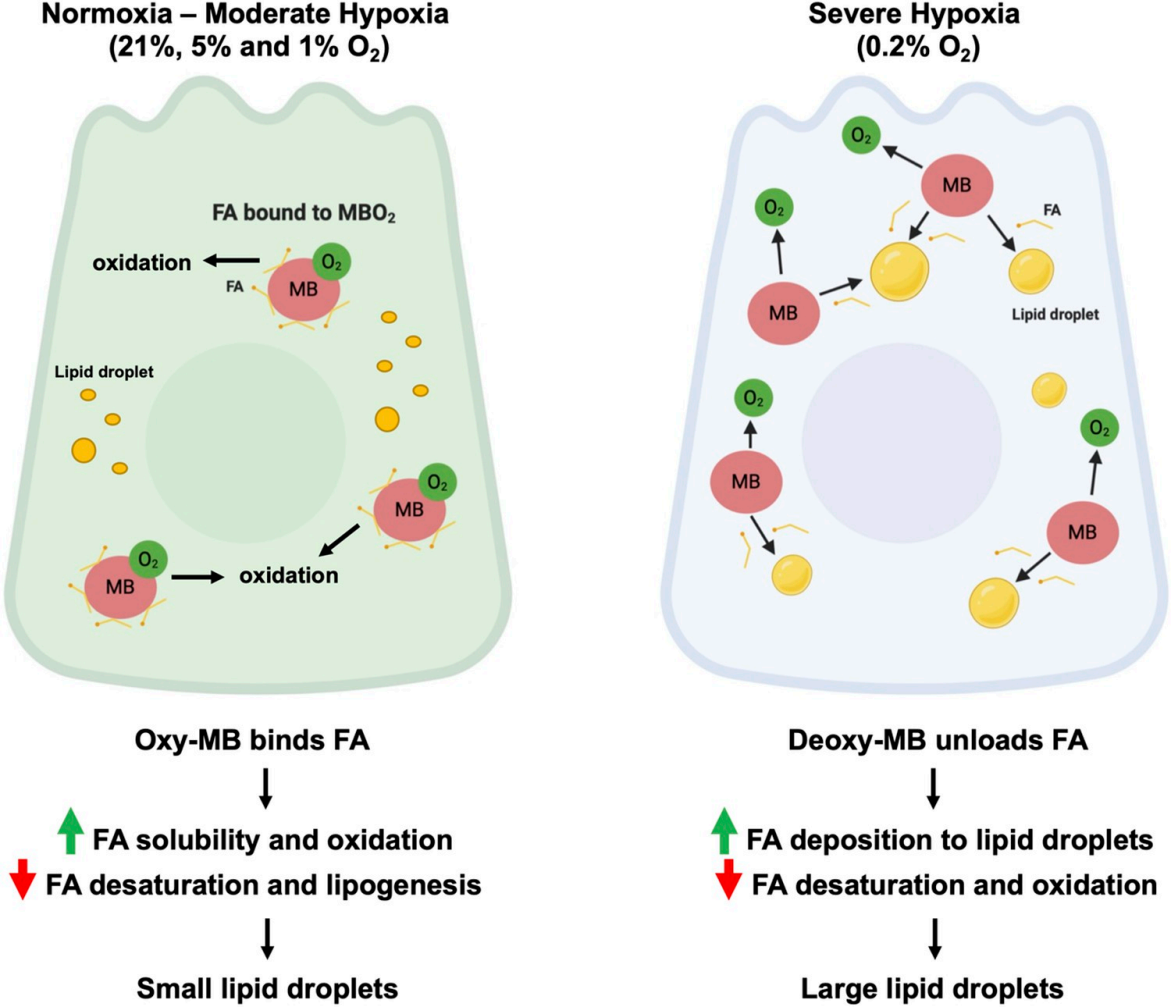
Model. Schematic diagram of MB’s role as an O_2_-dependent shuttle of FAs in mammary epithelial cells. Under normoxia and moderate hypoxia (left panel), oxygenated MB (MBO_2_) binds C16/C18 FAs and promotes cytoplasmic solubility and FA oxidation in association with smaller lipid droplets. Under severe hypoxia (right), MB unloads O_2_ and FA and deposits FAs to lipid droplets of larger sizes, potentially to prevent ROS poisoning, lipotoxicity and to store FA substrates for energy production upon reoxygenation.

Our current study revealed a novel function of MB in regulating FA metabolism in mammary epithelial cells. As summarized in Fig 6, MB expression not only promotes FA oxidation and antagonizes desaturation, but also increases FA cytoplasmic solubility when oxygen is available under normoxia and moderate hypoxia. During severe hypoxia, on the other hand, MB facilitates FA deposition to both LDs and lysosomes. Our findings in mammary epithelial cells are consistent with, and extend, previous observations of MB function in cardiomyocytes [17] and brown adipose tissue (BAT) [12].

### MB affects fatty acid composition and lipid droplets

FA profiles of mouse milk revealed that the luminal epithelia of the mammary gland produce a significantly higher proportion of medium chain FAs (C12:0, C14:0, and C14:1) in MB-proficient compared with MB-deficient tissues. On the other hand, levels of long-chain FAs (C16:0, C16:1n7c, and C18:1n9c) were significantly reduced in the milk of MBwt mice when compared to MBko counterparts. Hence, MB seems to promote *de novo* fatty acid synthesis and to stimulate the oxidative breakdown of longer chain FAs *in vivo*. In addition to impacting the entire turnover of FAs, presence of MB in mammary epithelial cells also appears to influence LD homeostasis through promoting FA cytoplasmic solubility and FA oxidation while antagonizing delta-9 desaturation, a rate-limiting step in lipogenesis. FA composition of mammary epithelial cells is known to control the size of intracellular and secreted LDs, specifically, a high abundance of palmitate promotes smaller LDs whereas oleate favours larger LDs [32]. Our current findings of MB-dependent regulation of LD homeostasis in mammary epithelial cells are consistent with previous observations by us and others that presence of oxygenated MB leads to numerous small LDs in BAT of mice [12, 13]. LDs accumulate in cancer cells under stress conditions, such as hypoxia [33, 34], nutrient starvation, and oxidative stress [35–38]. LDs, of different size, protein composition, spatial distribution, and lipid composition, can efficiently coordinate FA transport and consumption for energy production via mitochondrial ß-oxidation and the regulation of cellular redox state through FA uptake and release [39]. Sequestration of potentially oxidizable lipids such as cholesterols or ceramides into LDs is thought to prevent oxidative stress or to avoid lipotoxicity during subsequent reoxygenation events [40]. Our observation of an overlap between FAs and lysosomes under severe hypoxia is potentially relevant to the involvement of lysosomes in the LD breakdown through macrolipophagy. Following their transport to autophagosomes, LDs are degraded inside lysosomes using acidic lipases. The released FAs become available for energy production by mitochondrial ß-oxidation [33]. It is feasible that various pools of LDs exist in these cells, including resting LDs dedicated to storing FAs, along with other LDs that are involved in the macrolipophagic breakdown in lysosomes. This dynamic process appears to be facilitated by the O_2_-dependent trafficking and deposition of FAs through MB.

### MB is involved in fatty acid binding

Our current findings support the notion by Sriram et al. [41] that MB, through its palmitate-binding capacity, effectively competes with fatty acid binding proteins (FABPs) in transporting cellular FAs. Despite MB´s lower binding affinity for palmitate when compared with H-FABP (heart), MB-based FA shuttling might be favored due to MB’s high abundance (5 times more than H-FABP) and MB-mediated FA solubility. Hypoxia-driven LD biogenesis was previously shown to depend on the up-regulation of FABP3 and FABP7. Knockdown of these FABPs impaired LD accumulation during hypoxia and increased reactive oxygen species (ROS) levels in glioblastoma (U87 and T98G) and breast cancer (MDA-MB231 and MCF7) cells [36]. LD accumulation during hypoxia might serve to protect cells from free FA-driven lipotoxicity by storing FAs during oxygen deprivation and providing FAs for ATP production upon reoxygenation [36]. The same publication [36] showed that FABP3 and FABP7 are upregulated in MCF7 cells when exposed to severe hypoxia (0.1% O_2_) for 24–48 hours. Therefore, it is plausible that MB works in conjunction with FABP3 and FABP7 to deliver extracellular lipids into oxygen-deprived cells with inhibited lipogenesis.

### MB promotes fatty acid oxidation, not desaturation

MB expression in mammary epithelial cells appears to promote fatty acid oxidation while reducing stearyl-CoA desaturase (SCD) activity, a marker of lipogenesis. The latter is consistent with our recent observation that MB expression strongly decreased SCD1 mRNA expression in brown adipose tissue [12]. The relatively high abundance of the omega-9 FAs C16:1n9 (7-hexadecenoate or hypogeic acid) and C18:1n9 (oleate) in MDA-MB468 cells may be clinically relevant. As a product of limited oxidation of oleate, 7-hexadecenoate is a lipokine that is known to localize primarily in neutral lipids, to exert strong anti-inflammatory properties, and to be involved in phagocytosis *in vitro* and *in vivo* [29, 42], similar to omega-3 fatty acids. When mice are injected with 7-hexadecenoate, IL6 levels in the plasma were significantly reduced. Other inflammatory markers (IL12a, IL23a, TNF) were also down regulated in mouse peritoneal macrophage cells upon treatment with 7-hexadecenoate. Through activating limited oxidation of oleate to 7-hexadecenoate, oxygenated MB (MBO_2_) might be indirectly involved in controlling inflammatory signaling. Whether this impact is specific to breast cancer remains to be seen. Future research is needed to assess this clinically relevant potential by correlating MB expression to signalling molecules of inflammatory processes such as interleukins, leukotrienes, and NF-**Κ**B. If MB-affected FAs were found to potentially act as novel signalling cues (e.g., lipokines) in the differential progression of MB positive versus negative malignancies, antibodies directed against FAs might offer new therapeutic concepts to be combined with standard interventions of, for example, advanced metastatic breast cancer.

### MB expression may be essential for adaptation to hypoxic stress in cancer

Our group previously demonstrated that MB expression in epithelial carcinoma strongly correlates with hypoxia [19, 43, 44]. The MB protein expression was upregulated in cancer cells that are challenged by prolonged hypoxia and in peri-necrotic areas of ductal carcinoma in situ (DCIS), the *in vivo* model of tissue experiencing hypoxia. On the other hand, no hypoxic stimulation of MB expression was observed in normal epithelial cells. We and others also discovered that three novel alternatively spliced MB transcripts are predominantly expressed in breast cancer cell lines and breast tumors [19, 44]. Rather than being driven by the canonical promotor that is active in myocytic contexts, the cancer-specific expression of MB was under the control of a novel promoter [44], which is located 6 kb upstream of the ATG start codon and flanked by a functional hypoxia response element (HRE) to mediate hypoxic induction. Compared with the standard transcript in muscle tissues these transcript variants contain different non-coding 5-untranslated regions (5-UTRs), while the coding section contains the sequence for identical polypeptides in myocytes and breast epithelia. In the current study, we observed upregulated MB protein expression under severe hypoxia (0.2% O2 for 72 hours, S1B Fig) when MB is mostly deoxygenated [28] as an attempt to mimic long term hypoxia in tumors. It is important to note that hypoxia is not the only stimulator of MB expression in cancer cells or tissues. A transient but distinct induction of MB in human breast cancer cells was observed in response to mitogenic stimuli or oxidative stress. In parallel, the MB gene expression was found to be silenced by hormonal treatment in cancer cells [43, 44].

### MB as a potential intervention target and other future objectives

MB and other globins have been proposed as potential therapeutic targets in solid tumors and cancer cells [45]. Compared to the low-level expression of MB in the healthy breast epithelium, MB production in mammary malignancies can increase up to 350-fold [45]. Previous studies detected MB positivity in ~40% of primary breast tumors, mainly in a mosaic-like pattern in luminal-type, estrogen receptor (ER)-positive cases [10], and in ~53% of prostate cancer tumors, mostly in androgen-receptor positive and poorly differentiated entities [46]. Kaplan-Meier survival analyses of a large cohort of patients with mammary carcinoma associated high MB expression with beneficial prognostic outcomes for cases with positive or negative ERα receptor status [10]. Additionally, a trend towards prolonged recurrence-free patient survival was observed for MB-positive compared with MB-negative tumors in a cohort of poorly differentiated prostate tumors [46]. Our subsequent inspection of this apparent tumor-suppressive behavior exerted in vitro and in vivo by MB made use of CRISPR/Cas9-engineered MB-deficient breast cancer cells, in comparison to MB-proficient controls, and of spontaneous breast cancers, formed with and without MB, in two different mouse models (see: *a) Aboouf*, *M*.*A*., *et al*., *Pro-apoptotic and anti-invasive properties underscore the tumor suppressing impact of myoglobin on subset of human breast cancer cells*. *b) Aboouf*, *M*.*A*., *et al*., *Myoglobin Expression in Breast Cancer Mouse Models Reduces Metastasis and Tumorigenesis*. *Both manuscripts are currently under review*.*)*. These investigations revealed that the presence of MB in a subset of mammary carcinoma cells promotes apoptotic signaling and antagonizes cell motility as well as the partial epithelial to mesenchymal transition of the cells. Whether these pro-apoptotic, anti-invasive properties of MB are linked to its influence of the turnover and composition of FAs in normal and malignant breast epithelia reported above needs to be looked at by future work. Another future focus should analyze whether MB-positive carcinoma, in comparison with MB-deficient ones, exert different inflammatory activities in support of our proposed MB-driven production of anti-inflammatory cues. Adding substantiating evidence to this working hypothesis could mean that MB has the potential of being utilized as a stratified intervention target. As third main objective for upcoming work, MB’s control of FA turnover, composition and trafficking needs to be re-examined in relation to the physiology and pathology of myocytes and muscles. Lastly, additional studies are also needed to reveal the mechanisms of MB’s effect on the LD equilibrium/dynamics and FA metabolism in epithelial cells and to address the clinical implications of our discovery. Key questions remain: Do genetic mouse models of MB-deficient carcinomas differ from MB-positive tumor controls in vesicular structure or in LDs and associated FA composition? Can these findings in mice be translated into treatment of human cancer patients?

## Conclusions

The strengths of this work clearly lie in expanding the functional repertoire of an “old protein”. When expressed at high micromolar to millimolar concentrations, myoglobin (MB) is known to function as an oxygen delivery and storage protein in mammalian heart and skeletal muscles to support the cell´s high energy demands for contraction. However, the functions of endogenous MB in breast epithelia remain unclear where MB’s abundance is hundred-to-thousand-fold lower than that in myocytes. Here we report that, even at this low expression level, MB efficiently regulates fatty acid (FA) turnover, trafficking and composition in breast epithelia, at in vivo (milk) and in vitro (breast cancer cells) levels. By promoting the complete beta oxidation of FAs and limited oxidative remodeling, MB aids in supplying the cells not only with energy but also with new lipokine signaling cues that can potentially modulate inflammatory signaling cascade in these contexts (i.e., mammary carcinoma). Since presence of MB increased the cytoplasmic FA solubility under normoxia and FA deposition to lipid droplets under severe hypoxia, we now consider MB as O_2_-dependent shuttle of FAs, which agrees with previous in vitro studies assessing the loss of MB in cardiomyocytes. By depositing FA substrates to lipid droplets in severely O_2_-deprived epithelial cells, MB might aid in reducing lipotoxic insults during subsequent reoxygenation events. Together, the study at hand reveals the tight interplay between the functional repertoire of a protein and it´s abundance and/or expression site. This correlation has been largely overlooked in textbooks but hopefully will be considered more seriously in upcoming studies.

## Supporting information

S1 Fig Generation of MBko clones. DNA Sequencing and immunoblot analysis of MBko cells. (**A**) DNA and protein sequences of human *MB* exon 2 in wild-type allele and genetically altered alleles in MCF7 MB-KO cells. (**B**) Representative Western blot result showing the presence of MB (17 kDa) in wild-type cells and the absence of MB in the knockout (MB-KO) MCF7 cells, as well as MB upregulation under severe hypoxia. Cells were exposed to normoxia (Nx) or hypoxia (Hx, 0.2% O_2_) for 72 hrs before protein extraction. (**C**) Representative Western blot result to verify the MB knockout (MB-KO) in MDA-468 cells.
(TIF)

S2 Fig Lipid isolation. Thin layer chromatography (TLC) for the separation of different lipid groups. (**A**) Lipids extracted from the milk of MBwt and MBko mice. Each lane is loaded with lipids mixed with internal reference standards TG-17:0, DG-17:0, and PL-17:0 (10, 1, and 1 nmole/ml, respectively). These 17:0 unnatural fatty acids were co-purified following the Folch protocol and processed by transmethylation to allow for quantification by gas chromatography. (**B**) Lipids extracted from MBwt and MBko MDA-MB468 cells were mixed with TG-17:0, FFA-17:0, PL-17:0 and DG-17:0 internal reference standards (5, 5, 40, 10 nmole/mg protein, respectively). TG: Triglyceride, FFA: free fatty acid, DG: diglyceride, MG: monoglyceride, PL: phospholipid. MGs and DGs were combined for analysis of milk samples. Approximately 5–50 nmole of each standard were loaded to visualize the corresponding band on TLC.
(TIF)

S3 Fig Assessing MB-driven fatty acid distribution. **(A-C)** Cell staining. MBwt MDA-MB468 cells under severe hypoxia (Hx, 0.2% O_2_) were co-stained for BODIPY (green) and markers (red) of fatty acid importer (CD36) (**A**), peroxisomes (PMP70) (**B**) or mitochondria (MitoTracker) (**C**). The pictures shown are representative of 3 independent stainings. (**D**) MBwt and MB knockout (MBko) MCF7 cells were stained with BODIPY (green) prior to incubation to normoxia (Nx) or severe hypoxia (Hx, 0.2% O_2_) for 72 hrs before being co-stained for lysosome marker (Cytopainter red). The figures shown are representative of 4 independent experiments. Scale bar: 10 μm. Magnification: 63x. The signal intensity was measured by FACS as geometric mean (X-GMean, **E**). Students t-test was used for statistics (n = 6); mean ± SD.
(TIF)

## Abbreviations

BAT: brown adipose tissue
CRISPR/Cas9: clustered regularly interspaced short palindromic repeats / Crispr associated protein 9 endonuclease
FA: fatty acid
LD: lipid droplet
MB: myoglobin
MUFA: monounsaturated fatty acid
SFA: saturated fatty acid
TALEN: transcription activator-like effector nuclease
