# Correction: Myoglobin regulates fatty acid trafficking and lipid metabolism in mammary epithelial cells

**DOI:** 10.1371/journal.pone.0316741

**Published:** 2024-12-27

**Authors:** Julia Armbruster, Mostafa A. Aboouf, Max Gassmann, Angela Egert, Hubert Schorle, Veit Hornung, Tobias Schmidt, Jonathan L. Schmid-Burgk, Glen Kristiansen, Anne Bicker, Thomas Hankeln, Hao Zhu, Thomas A. Gorr

The affiliations of the seventh and eighth authors are incorrectly switched. Tobias Schmidt is not affiliated with #6 but with #7: Roche Pharmaceutical Research and Early Development, Roche Innovation Center Zurich, Schlieren, Switzerland. Jonathan L. Schmid-Burgk is not affiliated with #7 but with #6: Institute of Clinical Chemistry and Clinical Pharmacology, University and University Hospital Bonn, Bonn, Germany.
