# Supplementary figures and images for: Myoglobin regulates fatty acid trafficking and lipid metabolism in mammary epithelial cells
Source: PLoS One. 2022 Oct 12;17(10):e0275725. doi: 10.1371/journal.pone.0275725 (PMC9555620; doi:10.1371/journal.pone.0275725)

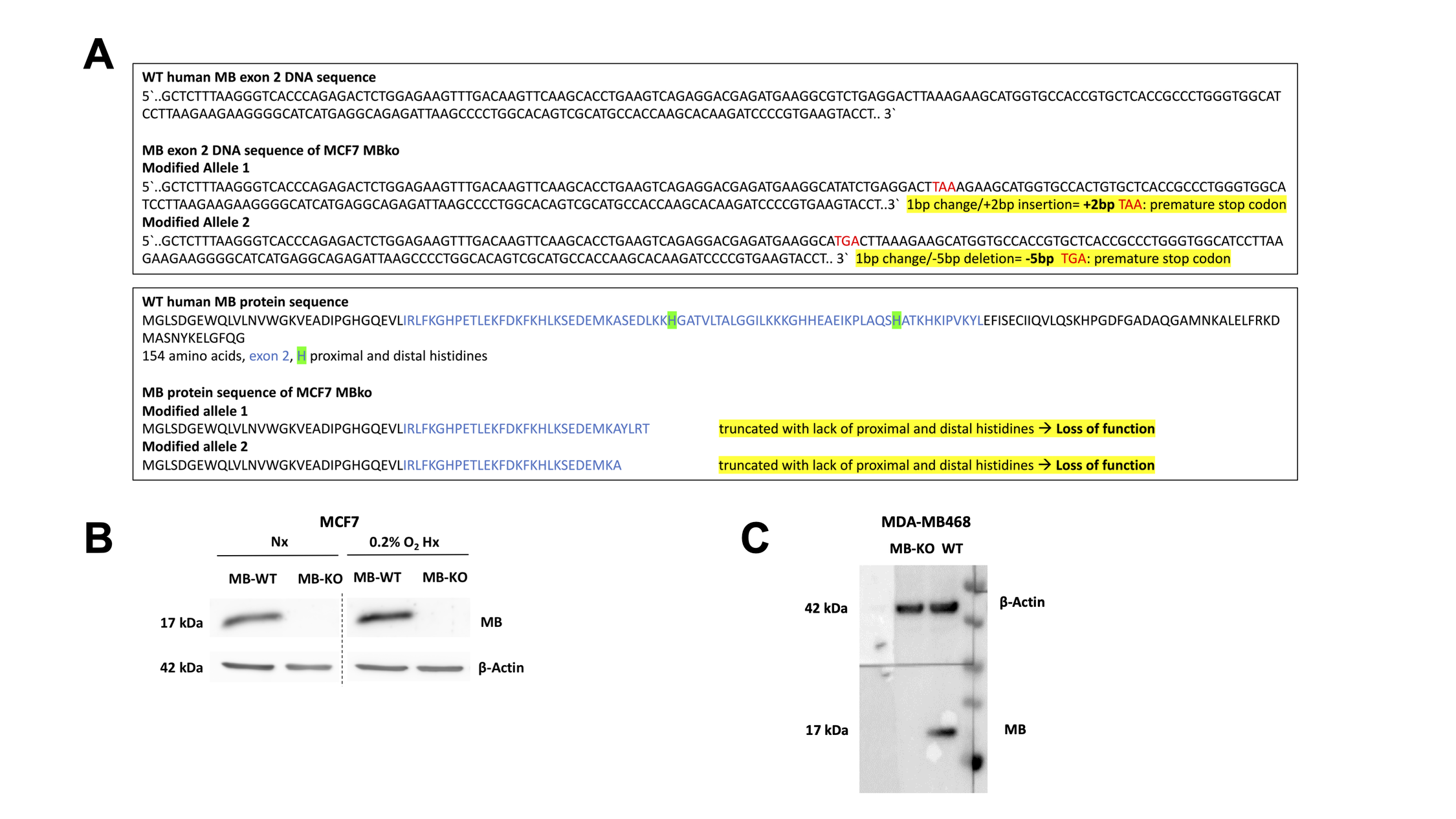

Supplement: S1 Fig — DNA Sequencing and immunoblot analysis of MBko cells. (A) DNA and protein sequences of human MB exon 2 in wild-type allele and genetically altered alleles in MCF7 MB-KO cells. (B) Representative Western blot result showing the presence of MB (17 kDa) in wild-type cells and the absence of MB in the knockout (MB-KO) MCF7 cells, as well as MB upregulation under severe hypoxia. Cells were exposed to normoxia (Nx) or hypoxia (Hx, 0.2% O2) for 72 hrs before protein extraction. (C) Representative Western blot result to verify the MB knockout (MB-KO) in MDA-468 cells. (TIF) [file pone.0275725.s001.tif]

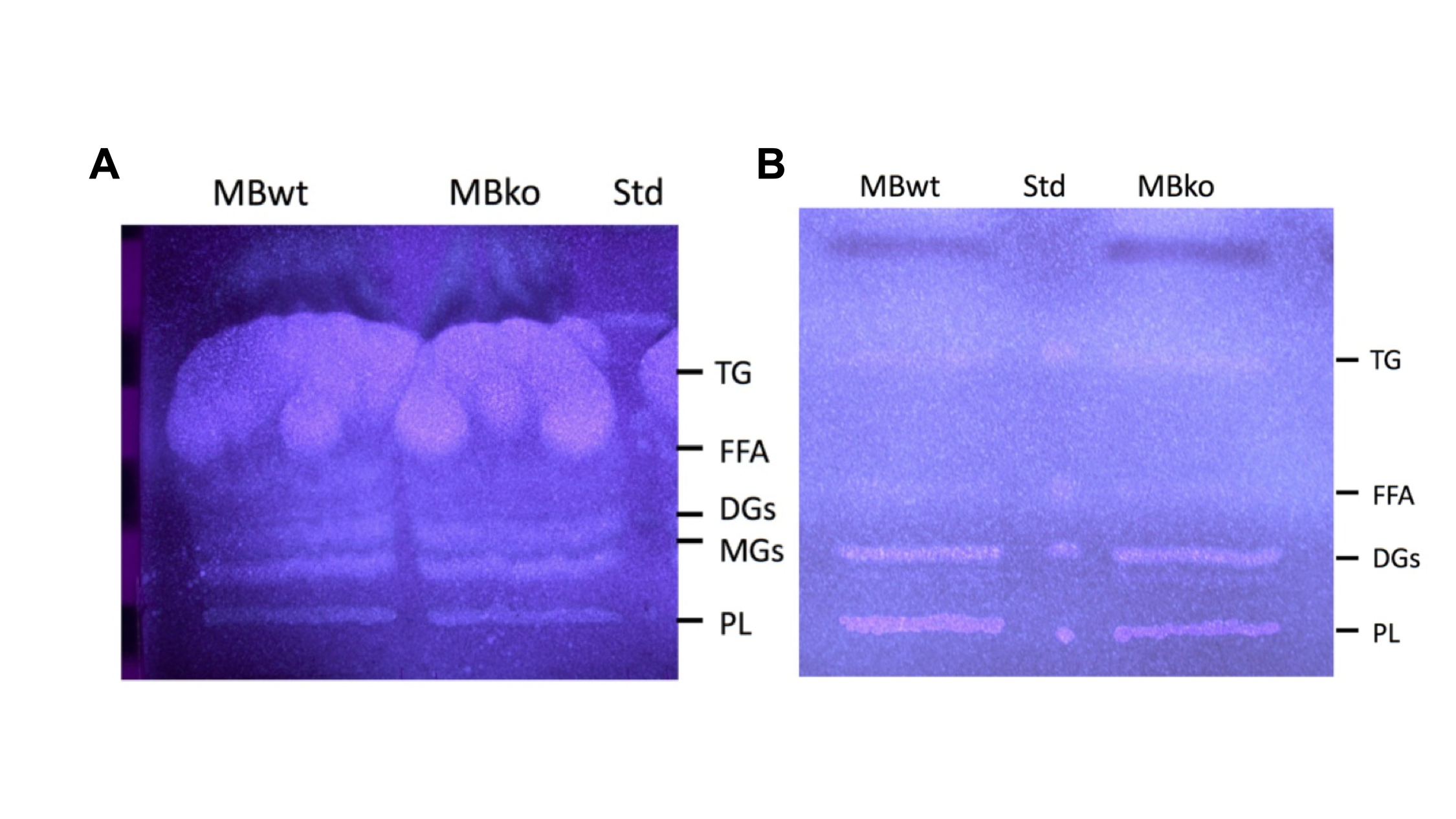

Supplement: S2 Fig — Thin layer chromatography (TLC) for the separation of different lipid groups. (A) Lipids extracted from the milk of MBwt and MBko mice. Each lane is loaded with lipids mixed with internal reference standards TG-17:0, DG-17:0, and PL-17:0 (10, 1, and 1 nmole/ml, respectively). These 17:0 unnatural fatty acids were co-purified following the Folch protocol and processed by transmethylation to allow for quantification by gas chromatography. (B) Lipids extracted from MBwt and MBko MDA-MB468 cells were mixed with TG-17:0, FFA-17:0, PL-17:0 and DG-17:0 internal reference standards (5, 5, 40, 10 nmole/mg protein, respectively). TG: Triglyceride, FFA: free fatty acid, DG: diglyceride, MG: monoglyceride, PL: phospholipid. MGs and DGs were combined for analysis of milk samples. Approximately 5–50 nmole of each standard were loaded to visualize the corresponding band on TLC. (TIF) [file pone.0275725.s002.tif]

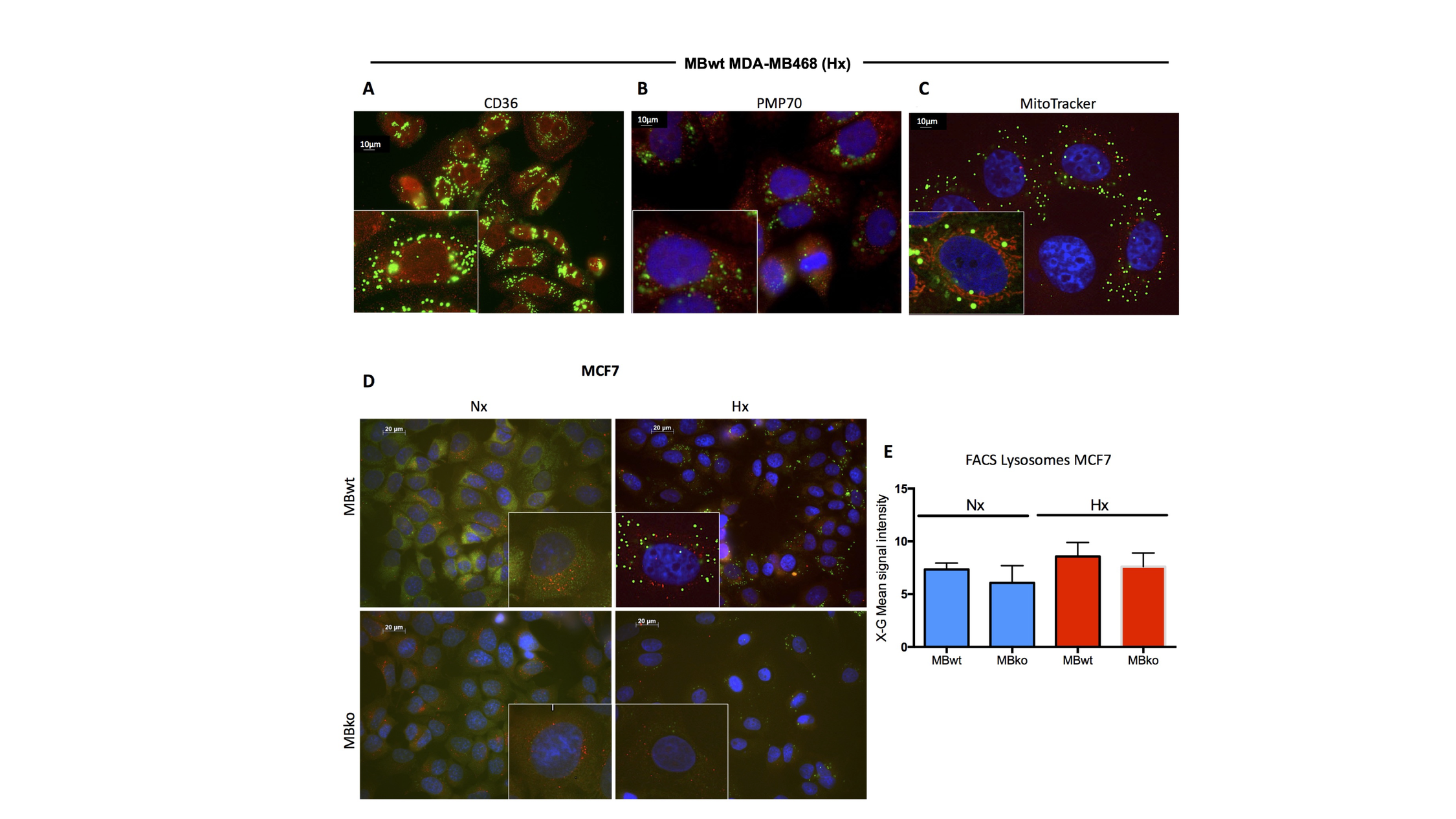

Supplement: S3 Fig — (A-C) Cell staining. MBwt MDA-MB468 cells under severe hypoxia (Hx, 0.2% O2) were co-stained for BODIPY (green) and markers (red) of fatty acid importer (CD36) (A), peroxisomes (PMP70) (B) or mitochondria (MitoTracker) (C). The pictures shown are representative of 3 independent stainings. (D) MBwt and MB knockout (MBko) MCF7 cells were stained with BODIPY (green) prior to incubation to normoxia (Nx) or severe hypoxia (Hx, 0.2% O2) for 72 hrs before being co-stained for lysosome marker (Cytopainter red). The figures shown are representative of 4 independent experiments. Scale bar: 10 μm. Magnification: 63x. The signal intensity was measured by FACS as geometric mean (X-GMean, E). Students t-test was used for statistics (n = 6); mean ± SD. (TIF) [file pone.0275725.s003.tif]
